# Reference Values for Minerals in Human Milk: the Mothers, Infants and Lactation Quality (MILQ) Study

**DOI:** 10.1016/j.advnut.2025.100431

**Published:** 2025-08-26

**Authors:** Lindsay H Allen, M Munirul Islam, Gilberto Kac, Kim F Michaelsen, Sophie E Moore, Maria Andersson, Janet M Peerson, Andrew M Doel, Daphna K Dror, Setareh Shahab-Ferdows, Daniela de Barros Mucci, Gabriela Torres Silva, Daniela Hampel, The MILQ Study Consortium, Christian Mølgaard, Sophie H Christensen, Jack I Lewis, Xiuping Tan, Daphna K Dror, Andrew M Doel, Bruna C Schneider, Farhana Khanam, Adriana Divina de Souza Campos, Fanta Nije, Mehedi Hassan, Amanda C Figueiredo

**Affiliations:** 1 Obesity and Metabolism Research, USDA, ARS Western Human Nutrition Research Center, Davis, California, United States; 2 Institute for Global Nutrition, Department of Nutrition, University of California, Davis, California, United States; 3 Nutrition Research Division, International Centre for Diarrhoeal Disease Research, Bangladesh (icddr,b), Dhaka, Bangladesh; 4 Nutritional Epidemiology Observatory, Josué de Castro Nutrition Institute, Federal University of Rio de Janeiro, Rio de Janeiro, Brazil; 5 Department of Nutrition, Exercise and Sports, Faculty of Science, University of Copenhagen, Copenhagen, Denmark; 6 Medical Research Council Unit The Gambia at London School of Hygiene & Tropical Medicine, Fajara, The Gambia, West Africa; 7 Department of Women and Children's Health, King's College London, London, United Kingdom; 8 Nutrition Research Unit, Children’s Research Centre, University Children's Hospital Zurich—Eleonore Foundation, Zurich, Switzerland; 9 Department of Basic and Experimental Nutrition, Rio de Janeiro State University, Rio de Janeiro, Brazil

**Keywords:** human milk, lactation, minerals, reference values, human milk nutrient concentration, infant mineral requirements, infant intakes

## Abstract

This fifth article in the series presenting reference values for nutrients in human milk describes minerals. The Mothers, Infants and Lactation Quality (MILQ) and Early-MILQ studies collected human milk samples throughout the first 8.5 mo of lactation in 1242 well-nourished women in Bangladesh, Brazil, Denmark, and The Gambia. All minerals were measured by inductively coupled plasma-mass spectrometry. Although pooled MILQ medians from 1 to 6 mo are within ∼10% of the concentration used by the Institute of Medicine (IOM) for magnesium, potassium, calcium, and copper, they are ∼50% of the IOM value for zinc and selenium, and ∼75% of the IOM value for sodium and iron. For zinc, sodium, and iron, the discrepancy can be explained by the IOM’s use of values from early lactation (<3 mo) when the milk nutrient concentrations are higher; in contrast, for potassium the IOM benchmark concentration is consistent with later lactation (6 mo) in MILQ. Pooled median MILQ phosphorus from 1 to 6 mo is 120% of the concentration selected by the IOM. Milk iodine concentrations in MILQ varied among sites, reflecting the differing national policies for salt iodization. Total daily median mineral intakes from 1 to 6 mo were 49%–55% of IOM adequate intakes (AIs) for zinc and selenium, 74%–90% of AIs for sodium, iron, and magnesium, and 110%–125% of AIs for copper, potassium, calcium, and phosphorus. For zinc, sodium, iron, and potassium, differences can be explained by the reference time frame during lactation. The MILQ study mineral concentrations are provided as percentile curves to enable comparison and interpretation. Importantly, the MILQ data show marked changes in milk mineral concentrations during the first 6 mo of lactation, an observation often missed because of the absence of data representing a spectrum of time postpartum in previously published data.

**Statement of Significance**

This paper from the series on reference values (RVs) for nutrients in human milk describes concentrations of multiple minerals across the first 8.5 mo of lactation, and because milk volumes were measured at the same times that milk was collected, the total intakes of multiple minerals. The study design enabled modeling of concentrations, by value and percentile, across time postpartum, such that the timing of the large changes in some minerals is documented and can better inform recommendations for mineral intakes of infants during the periods of change.

## Introduction

This article is the fifth in a series of 7 in this journal supplement, which describes the process and outcomes of the Mothers, Infants and Lactation Quality (MILQ) Study. The MILQ study was designed to develop reference values (RVs) for nutrient concentrations in the milk of healthy mothers. The study design and methods have been described in detail previously [1]. In brief, human milk samples and anthropometric, dietary, biochemical, and other data were collected from mother–infant dyads at 4 study sites (Bangladesh, Brazil, Denmark, and The Gambia). Collection occurred from 0 to 1 mo [early milk, or E-MILQ, with sample collection at 4–17 d (E1) and 18–31 d (E2)], then 3 times between 1 and 8.5 mo lactation [1–3.49 mo (M1), 3.5–5.99 mo (M2), and 6–8.5 mo (M3)]. The RVs are presented as pooled median concentrations and percentiles. The other articles in this journal supplement are an introduction, milk volumes, and RVs for macronutrients, water-soluble vitamins, and fat-soluble vitamins. The supplement concludes with an article on the applications of the research.

Minerals comprise a broad category of inorganic substances, including essential trace elements. In the human body, minerals have structural roles in bone and teeth, regulate body fluids, support nerve and muscle function, and act as cofactors for enzymes essential for physiological processes. Although a deficiency in some minerals early in life is associated with infections, disease, and compromised growth [2,3], an excess can be toxic [4].

Given their roles in health and disease, accurate quantification of minerals in human milk is paramount for understanding infant and maternal requirements during breastfeeding. Existing adequate intake (AI) recommendations are based on data from outdated studies, often involving few participants, inconsistent human milk collection methods, and analytical methodologies that did not account for the unique qualities of the milk matrix [5].

We have recently validated an inductively coupled plasma-mass spectrometry (ICP-MS) method for simultaneously and accurately measuring trace elements and minerals in human milk using acid digestion [6]. Within 1 analysis, it is possible to quantify multiple minerals present in a wide range of concentrations using small milk volumes. With the exception of iodine, 3095 samples were analyzed (2520 MILQ, 575 E-MILQ), of which 314 were removed for not meeting inclusion criteria (262 MILQ, 52 E-MILQ). The most common reasons for sample exclusion were infant anthropometric *Z*-scores < −2.0, maternal factors, or cessation of exclusive breastfeeding (EBF) through visit M1 [7]. For iodine, we used an ICP-MS method based on alkaline digestion [8]. The benefits of ICP-MS include low detection limits, low sample volume, simple sample preparation, and high sample throughput.

MILQ and E-MILQ results are compared with currently accepted values [mostly those accepted by the Institute of Medicine (IOM) (renamed National Academy of Medicine (NAM) in 2015) for deriving intake recommendations] and those reported elsewhere, including the recent data from samples collected from 2007 to 2011 in the large (*n* = 559–835) Canadian Maternal-Infant Research on Environmental Chemicals (MIREC) pregnancy cohort [9]. Sample collection in MIREC was from 3 wk to 2.5 mo, so comparisons with later MILQ samples were not possible. Older nutrient reference concentrations should be accepted with caution because of possibly inaccurate analytical methods.

The graphs provided in this manuscript show the median concentration of each nutrient in milk by site and day of lactation with individual data points in the background and the modeled 5th, 10th, 25th, 50th, 75th, 90th, and 95th percentiles by day of lactation. Estimated percentile curves were constructed using generalized additive models for location, scale, and shape (GAMLSS) [10] and the *GAMLSS* package (V5.4-20) using age (in days) as the only explanatory variable. Total daily intakes of each mineral were calculated as concentration × milk volume for each mother–infant dyad at each time point. A detailed description of methods used to quantify milk volume can be found elsewhere [7]; in brief, volume was measured over 14-d periods using the stable isotope dilution dose-to-mother method except in MILQ in Denmark, where before- and after-test weighing was used. Comparisons with IOM nutrient reference concentrations and AIs are presented in TABLE 1, TABLE 2. Supplemental tables 1 and 2 include estimated percentile values of nutrient concentration by month postpartum and median total nutrient intake by study visit (1–3.49 mo, 3.5–5.99 mo, 6–8.5 mo).

**TABLE 1:** Comparison of MILQ (1–6 mo) and IOM (NAM for sodium and potassium) values for mineral concentrations in human milk.

Nutrient	MILQ^1^ Median	MILQ IQR	IOM	MILQ median as % IOM
Sodium (mg/L)	111	(87–156)	140^2^	79
Potassium (mg/L)	542	(485–609)	515	105
Magnesium (mg/L)	33	(28–37)	34	97
Phosphorus (mg/L)	150	(133–169)	124	121
Calcium (mg/L)	287	(249–325)	259	111
Iron (mg/L)	0.25	(0.18–0.34)	0.35^2^	71
Copper (mg/L)	0.26	(0.20–0.34)	0.25	104
Zinc (mg/L)	1.29	(0.84–1.78)	2.5^2^	52
Selenium (μg/L)	9	(7–11)	18	50

Abbreviations: IOM, National Academy of Medicine; MILQ, Mothers, Infants, and Lactation Quality Study; NAM, National Academy of Medicine.

1 MILQ values are pooled median concentrations from 1 to 6 mo.

2 The IOM reference time frame for these minerals is early lactation (<3 mo) when concentrations are higher than at 3–6 mo.

**TABLE 2:** Comparison of MILQ pooled median mineral intakes in exclusively breastfed infants from 1 to 6 mo with IOM (NAM for sodium and potassium) adequate intakes.

Nutrient	MILQ median	MILQ IQR	IOM AI	MILQ median as %AI
Sodium (mg/d)	96	(71–127)	110	87
Potassium (mg/d)	452	(381–531)	400	113
Magnesium (mg/d)	27	(22–32)	30	90
Phosphorus (mg/d)	125	(103–149)	100	125
Calcium (mg/d)	239	(192–290)	200	120
Iron (mg/d)	0.20	(0.15–0.28)	0.27	74
Copper (mg/d)	0.22	(0.17–0.28)	0.20	110
Zinc (mg/d)	1.1	(0.7–1.5)	2.0	55
Selenium (μg/d)	7.3	(6.0–9.2)	15.0	49

Abbreviations: AI, adequate intake; IOM, National Academy of Medicine; MILQ, Mothers, Infants, and Lactation Quality Study; NAM, National Academy of Medicine.

## Sodium

### Background

Sodium is an essential mineral that maintains fluid and mineral balance and plays a role in muscle function and nerve impulse transmission. Deficiency is associated with neurological consequences and poor growth [11]. Infancy may be a sensitive period with respect to the influence of excess sodium intake on the future risk of hypertension [12]. However, there is no clear correlation between maternal sodium dietary intake and human milk sodium concentration [13]. The milk of mothers who deliver prematurely has a higher sodium concentration than that of mothers who deliver at term [11]. Sodium concentrations in milk are influenced by milk osmolarity, particularly the concentration of lactose [14]. As discussed below, sodium concentrations in milk increase in mastitis, and the sodium:potassium ratio has been used as a biomarker for this condition.

### Results

**FIGURE 1:**
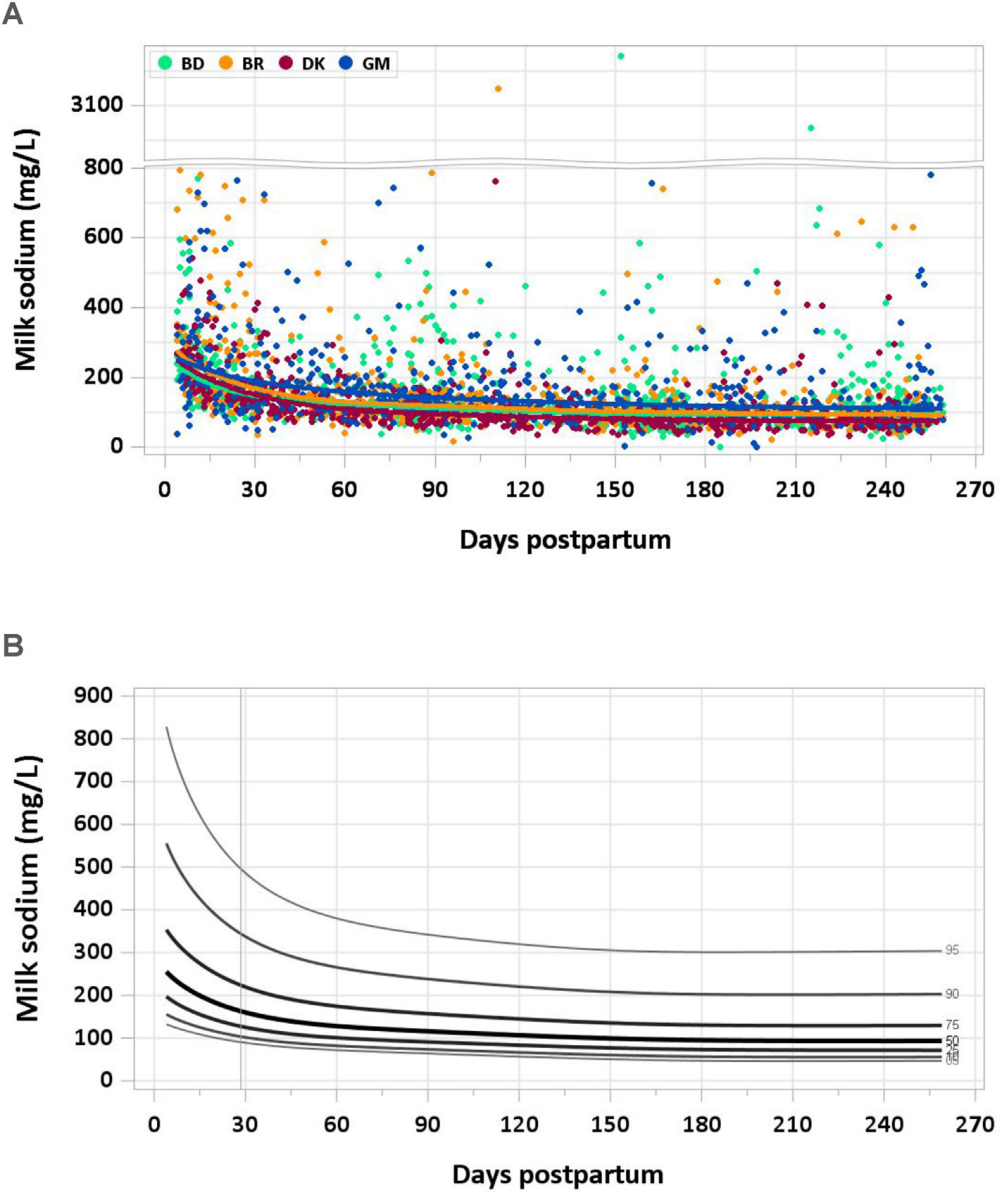
(A) Distribution of human milk sodium concentrations. (B) Percentile curves for sodium concentration in human milk.

No samples were excluded because of implausible sodium concentrations. The median milk sodium concentration was highest at 4–17 d (228 mg/L) and decreased to 140 mg/L at 1–2 mo. Thereafter*,* it continued to fall gradually to a median of 111 mg/L at 3–4 mo and 97 mg/L at 5–6 mo (Figure 1A and B). The pooled median total sodium intake from 1 to 6 mo was 96.5 mg/d.

### Comparison with published values

The NAM revised the AI for sodium for infants 0–6 mo to 110 mg/d in 2019, based on an average human milk sodium concentration of 140 mg/L determined from a review of 20 studies and an average milk consumption of 0.78 L/d [15]. Although the NAM chose to pool data, in the referenced studies sodium concentrations tended to be >140 mg/L when milk was collected early in lactation and <140 mg/L in studies where milk was collected later (3–6 mo) [14]. In the Canadian MIREC study, the 50th percentile was 148 mg/L (152 μg/g) from 3–10 wk when concentrations are known to be higher than at 5–6 mo. Milk sodium concentrations in the MILQ study generally agree with the MIREC results and the NAM data but suggest that the NAM value used to set the AI may overestimate milk sodium concentration after 3 mo of lactation.

## Potassium

### Background

Potassium is the principal cation in intracellular fluids and is essential for maintaining membrane potential and other vital biological roles, including muscle contraction and nerve function. Infants must maintain a positive potassium balance to sustain somatic growth [16]. Potassium is widely distributed in the food supply, and deficiency is uncommon unless an illness causes malabsorption or excessive excretion.

### Results

**FIGURE 2:**
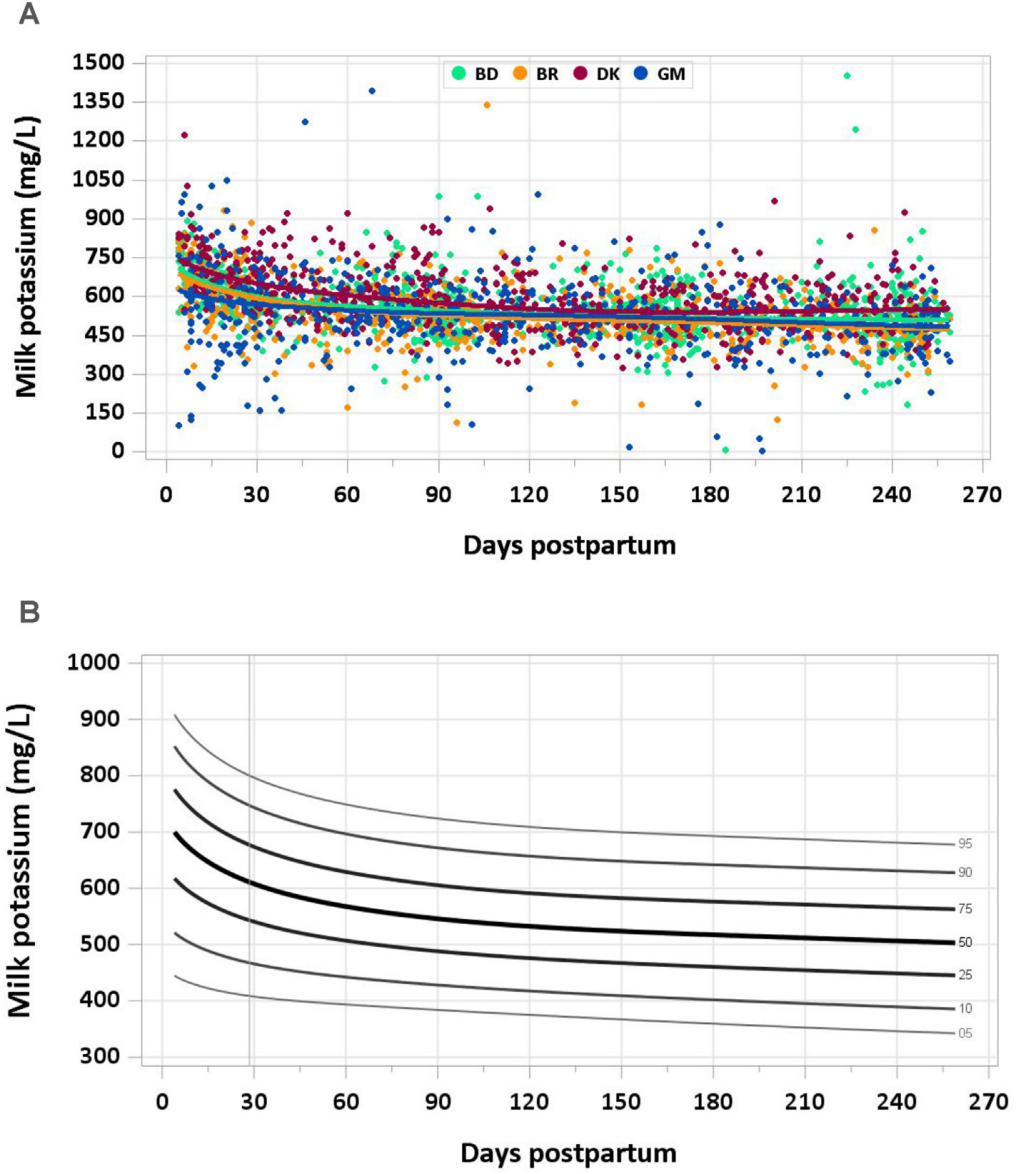
(A) Distribution of human milk potassium concentrations. (B) Percentile curves for potassium concentration in human milk.

No samples were excluded from the analysis for implausible potassium concentrations. Milk potassium concentration decreased most rapidly in the first month of lactation from a median of 659 mg/L at 4–17 d to 584 mg/L at 1–2 mo and continued to decline gradually through 8.5 mo postpartum. At 5–6 mo, the pooled median milk potassium concentration was 520 mg/L (Figure 2A and B). The pooled median total potassium intake from 1 to 6 mo was 452 mg/d.

### Comparison with published values

The NAM AI value for potassium for infants 0–6 mo is 400 mg/d, based on an average human milk potassium concentration of 515 mg/L from 17 studies and an intake of 0.78 L/d [15]. The declining trend in milk potassium concentration throughout lactation is consistent with trends demonstrated in longitudinal data included in the NAM’s report on potassium, published in 2019 [15]. Results of the present study suggest that the NAM concentration used for 0–6 mo may underestimate that of human milk earlier in lactation but is generally consistent with the median concentration through 6 mo. The milk potassium concentration of 566 mg/L (583 μg/g) in the MIREC cohort likely reflects the earlier collection of samples (3–10 wk) [9].

## Sodium:potassium ratio

### Background

The Na:K molar ratio is commonly used as an indicator of subclinical mastitis, an inflammation of the mammary gland that is not clinically symptomatic but is marked by an increased milk concentration of the inflammatory cytokine IL-8. Data are presented here because this is a unique opportunity to describe the ratio over time postpartum in 4 locations with different sanitary environments—but an RV cannot be estimated. In a 2022 study of milk samples from Japanese women with and without clinical mastitis, a Na:K ratio > 0.6 was considered an optimal diagnostic cut-off using the ion-selective electrode method [17]. Other investigators found that a higher Na:K ratio cut-off of 1.0–1.1 was more consistent with the incidence of clinical mastitis [18]. The percentage of subclinical mastitis cases that resolve without developing into clinical mastitis is unknown. Aside from being a biomarker for mastitis, an elevated Na:K ratio (> 0.80) at 7 d postpartum may predict a delay of second-stage lactogenesis, poor milk supply, and early breastfeeding cessation [19].

### Results

**FIGURE 3:**
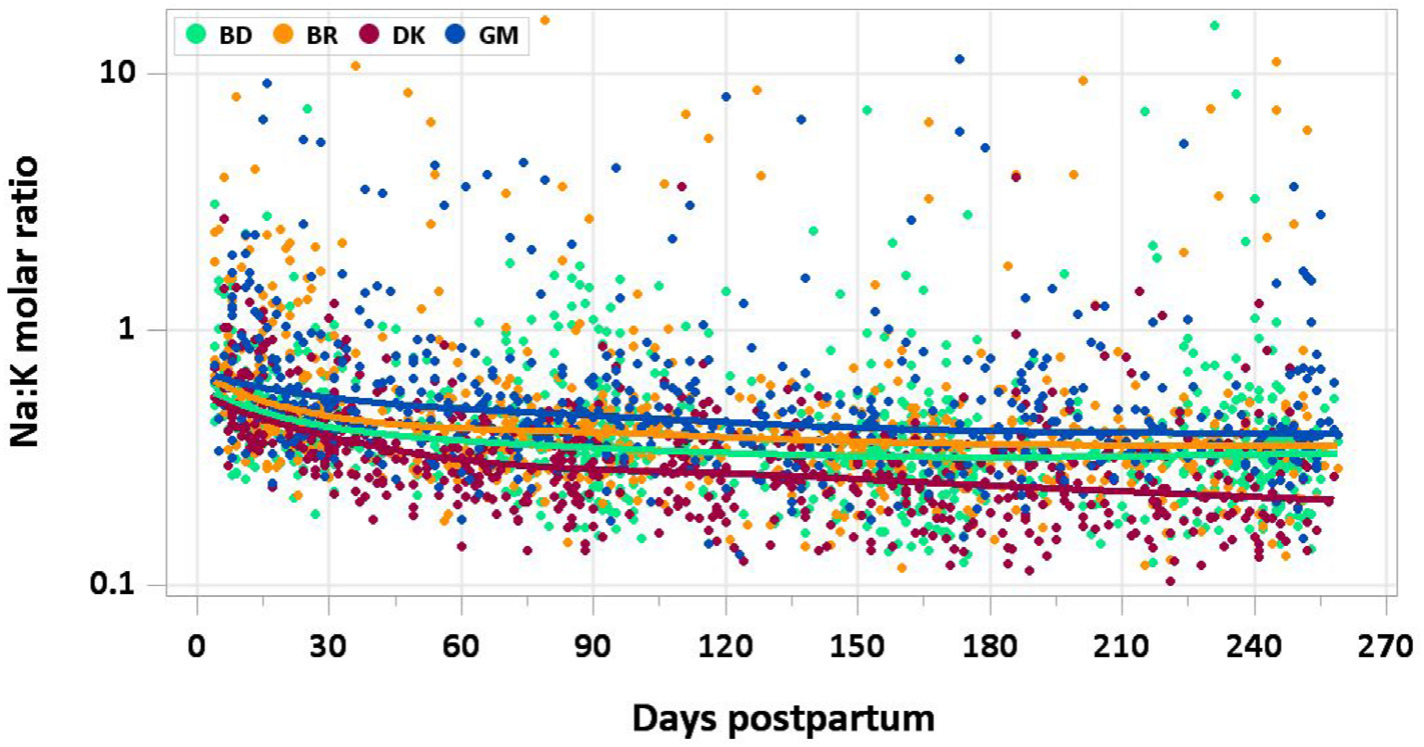
Distribution of human milk Na:K ratio.

In the E-MILQ and MILQ studies, median Na:K molar ratios were 0.51 and 0.35, respectively. In MILQ, the prevalence of an elevated Na:K ratio (>0.6) was 13% in Bangladesh, 14% in Brazil, 4% in Denmark, and 25% in The Gambia (Figure 3). Prevalences were much higher during the first month of lactation (E-MILQ): 28% in Bangladesh, 47% in Brazil, 29% in Denmark, and 49% in The Gambia.

### Comparison with published values

There are no published values that can be used for comparison.

## Magnesium

### Background

Magnesium is involved in energy production, nucleic acid and protein synthesis, ion transport, and cell signaling. It is stored in the human body primarily in bone. During lactation, maternal dietary intake is the primary source of magnesium, whereas bone mobilization contributes small amounts [20]. Magnesium is widely distributed in foods and is more concentrated in seeds and legumes than in animal source foods. Deficiency is rare, although status is difficult to measure. Little is known about the effects of maternal diet or supplements on milk magnesium, although such effects are unlikely [21].

### Results

**FIGURE 4:**
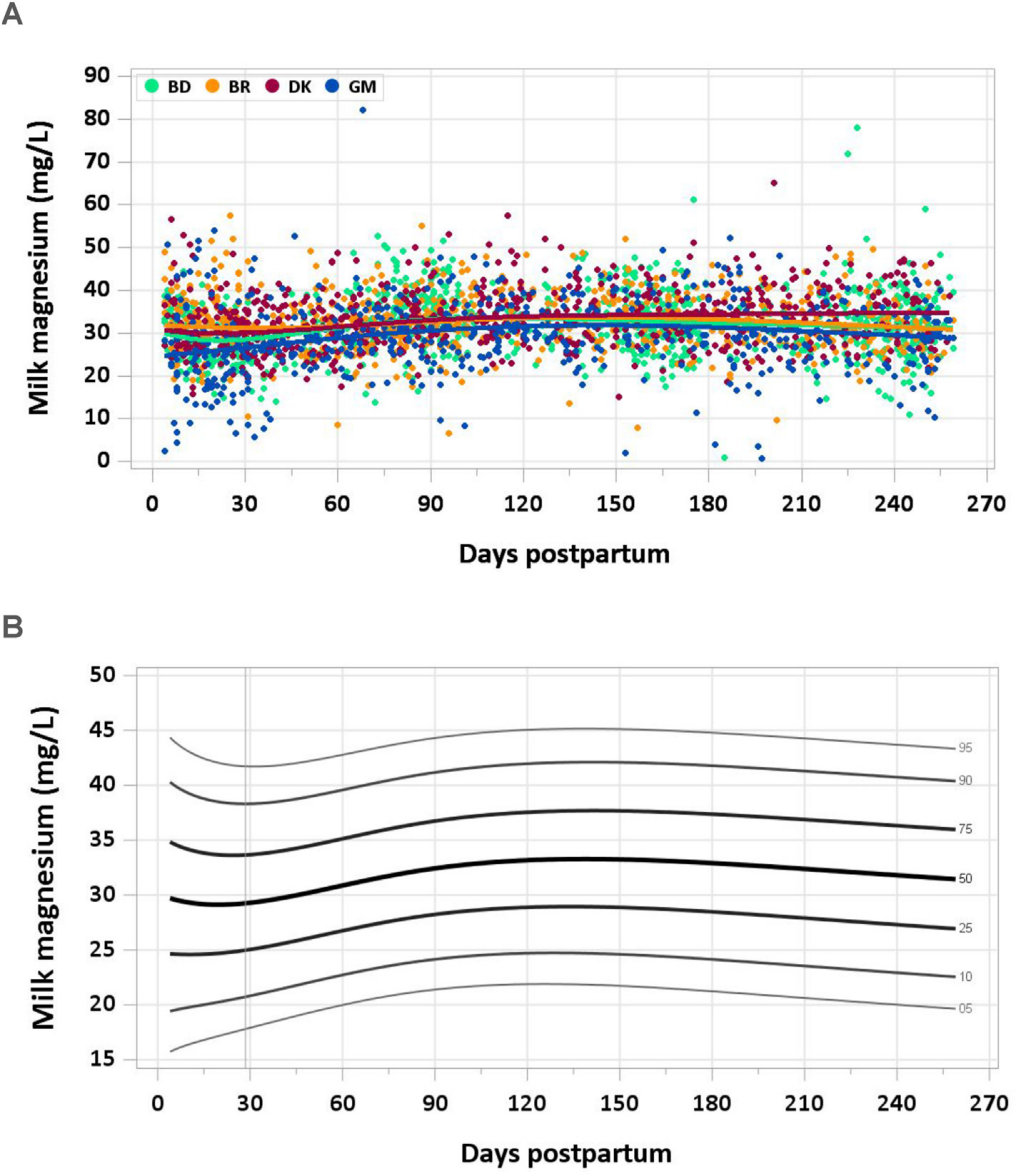
(A) Distribution of human milk magnesium concentrations. (B) Percentile curves for magnesium concentration in human milk.

No samples were excluded from the analysis because of implausible magnesium concentrations. Milk magnesium was stable during lactation, with pooled median concentrations of 29.4 mg/L at 4–17 d, 30.1 mg/L at 1–2 mo, and 31.6 mg/L by the end of the study period (Figure 4A and B). The pooled median total magnesium intake from 1 to 6 mo was 27 mg/d.

### Comparison with published values

According to the IOM, the AI for magnesium from 0 to 6 mo is 30 mg/d based on an estimated human milk magnesium concentration of 34 mg/L and an average milk intake of 0.78 L/d. This intake allows for a positive magnesium balance of ≥10 mg/d in early infancy [22]. The milk magnesium concentrations in the MILQ study are close to the IOM concentration used to set the AIs and on par with a review of magnesium in human milk, which found a median concentration of 31 mg/L [23]. The Canadian MIREC study also reported milk magnesium concentration as 31 mg/L (32 μg/g) from 3 to 10 wk [9].

## Phosphorus

### Background

Phosphorus serves as a structural component of cell membranes and nucleic acids and is involved in a variety of biological processes, including bone mineralization, cell signaling, energy production, and regulation of acid–base balance. Phosphorus concentration in human milk is tightly regulated [24], and although regulation mechanisms are distinct, there is a ratio between calcium and phosphorus concentrations of ∼2:1 [25,26]. A 1999 review of 37 studies concluded that milk phosphorus concentration is not influenced by maternal dietary intake, age, parity, lactation history, method of sampling, or smoking [25].

### Results

**FIGURE 5:**
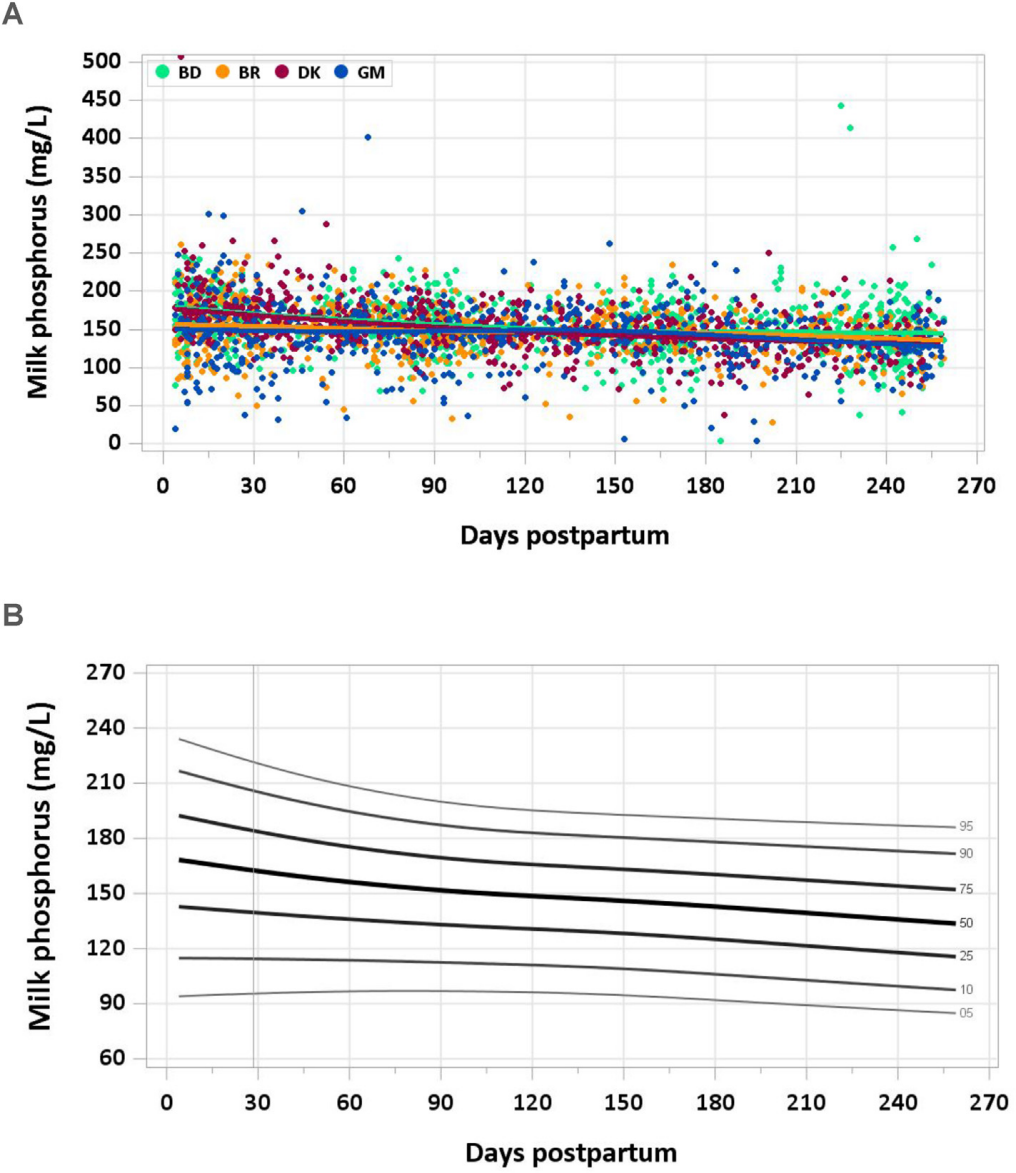
(A) Distribution of human milk phosphorus concentrations. (B) Percentile curves for phosphorus concentration in human milk.

No samples were removed from the analysis because of implausible phosphorus concentrations. There was a steady decline in milk phosphorus concentration across lactation, from a median of 165 mg/L in the first month to 144 mg/L at 5–6 mo and 135 mg/L at the end of the study period (Figure 5A and B). The pooled median total phosphorus intake from 1 to 6 mo was 125 mg/d.

### Comparison with published values

The IOM has set the AI for phosphorus at 100 mg/d based on 2 studies reporting an average milk phosphorus concentration of 124 mg/L after the first month of lactation [27,28] and an estimated milk intake of 0.78 L/d. This AI is associated with a circulating phosphorus concentration that does not result in excess renal excretion [22]. The milk phosphorus concentrations measured in the MILQ study are ∼15%–30% higher than the IOM reference concentration and on par with a review suggesting a median milk phosphorus concentration of 143 mg/L [29]. The MIREC study from Canada reported a mean concentration of 138 mg/g (143 μg/g) from 3 to 10 wk [9]. Thus, the IOM value may be low.

## Calcium

### Background

Calcium is a principal constituent of bone, providing strength and structure to the skeleton, and plays a critical role as a messenger in cell-signaling pathways. Human milk calcium is tightly linked with casein and citrate [30]. Some published data indicate that maternal dietary calcium intake can impact human milk concentrations, especially when habitual consumption is low [31,32]. To ensure an adequate infant supply to support growth, milk calcium concentrations are maintained by homeostatic mechanisms. Maternal bone mineral mobilized during early lactation is recovered postweaning, and successive or long lactation periods are not associated with progressive bone loss [33].

### Results

**FIGURE 6:**
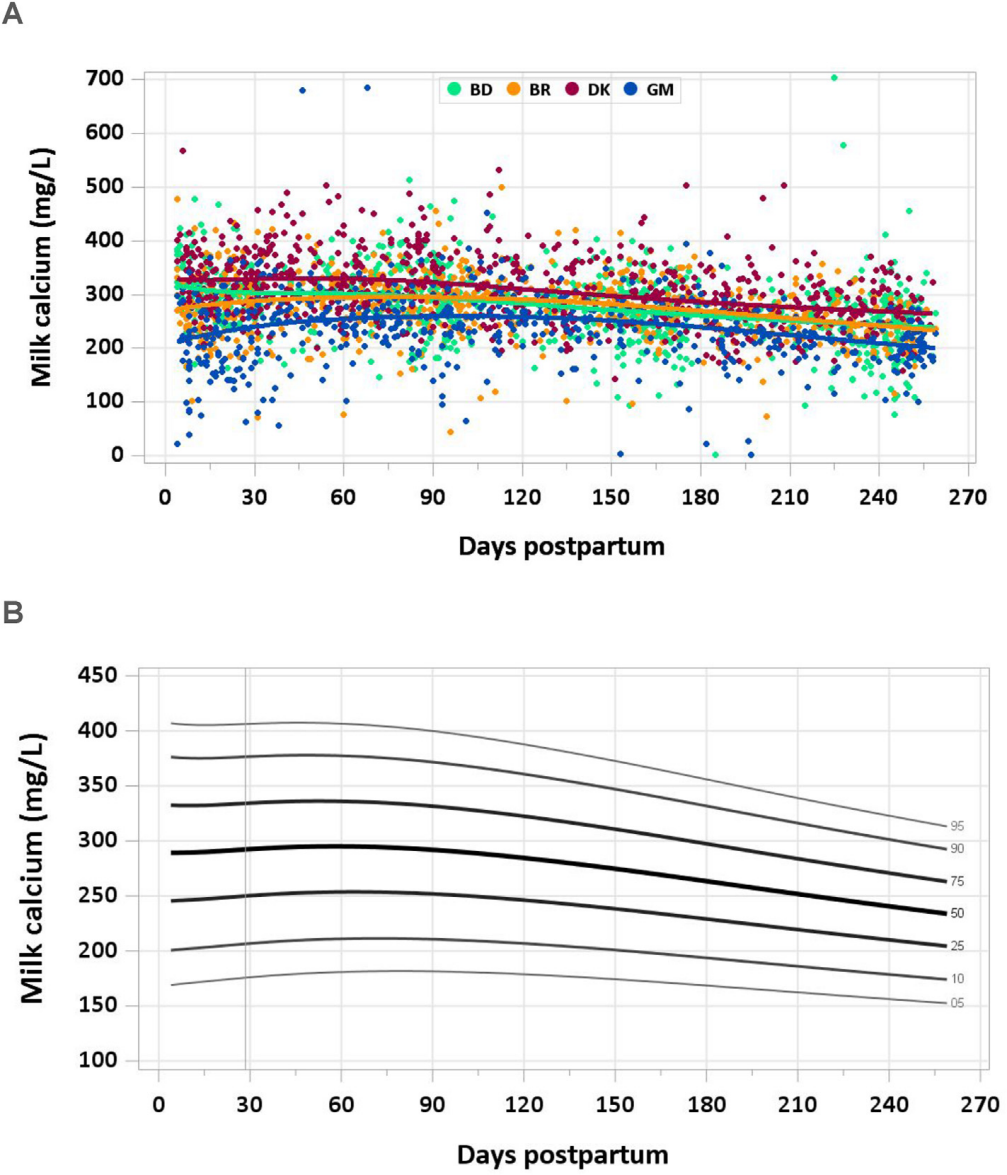
(A) Distribution of human milk calcium concentrations. (B) Percentile curves for calcium in human milk.

No samples were excluded for implausible concentrations of milk calcium. Milk calcium concentrations decreased gradually throughout lactation, from a median of 329 mg/L at 4–17 d to 294 mg/L at 1–2 mo, 268 mg/L at 5–6 mo, and 237 mg/L at the end of the study period (Figure 6A and B). The pooled median total calcium intake from 1 to 6 mo was 239 mg/d.

### Comparison with published values

The AI for calcium in infants 0–6 mo of age is 200 mg/d, which reflects an estimated calcium concentration of 259 ± 59 mg/L in breastmilk and a milk intake of 0.78 L/d [34]. Milk calcium concentrations in the MILQ study were similar to the IOM value. A recent meta-analysis of 154 studies on human milk calcium from 0 to 36 mo lactation found a mean concentration of 262 mg/L [95% confidence interval (CI): 243, 282] in samples from all women and 249 mg/L (95% CI: 232, 266) in studies of healthy women with healthy term infants who were EBF [35]. In this same meta-analysis, milk calcium concentration over the first year of lactation exhibited a gradual decrease that could be modeled linearly. Calcium concentrations in the MIREC study were substantially higher [315 mg/L (325 μg/g) from 3 to 10 wk] than IOM or MILQ values, likely related to the collection of samples earlier in lactation [9]. The higher milk calcium in Denmark in the MILQ study suggests that a higher usual maternal calcium intake, including supplementation during pregnancy, might increase the concentrations in milk.

## Iron

### Background

Iron plays a critical role as a component of many proteins, including hemoglobin and enzymes involved in energy production. Infants are particularly vulnerable to the consequences of iron deficiency because of their rapid growth and brain development [36]. A healthy full-term newborn has hepatic iron reserves that can meet its needs through 4–6 mo of age when hematopoiesis is active and exogenous sources of iron are available [37]. It is well recognized that although present at low concentrations, iron in human milk is highly bioavailable to the infant [38]. Neither maternal iron status nor maternal supplementation affects milk iron concentrations [39]. The sample preparation methods used in the MILQ study were effective at releasing iron bound to low-molecular-weight peptides, fat globules, and lactoferrin in milk.

### Results

**FIGURE 7:**
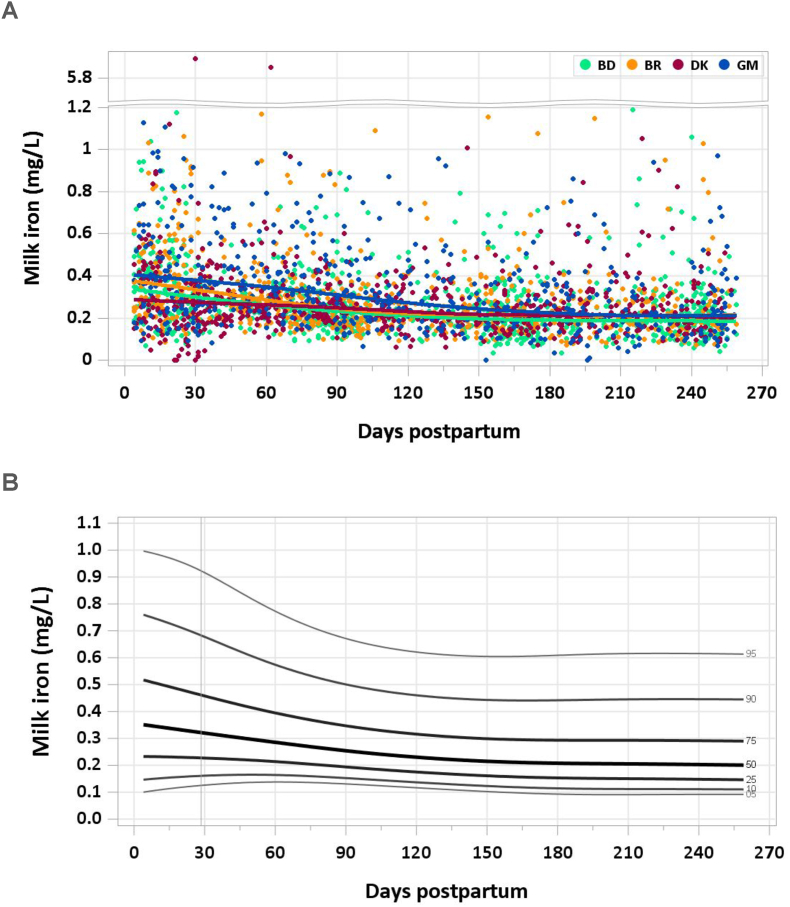
(A) Distribution of human milk iron concentrations. (B) Percentile curves for iron concentration in human milk.

Two samples were excluded from the analysis for implausible concentrations of milk iron (> 6 mg/L) in each of the MILQ and E-MILQ sample sets and 8 samples for implausible negative values in the E-MILQ set. The concentration decreased from a median of 0.38 mg/L at 4–17 d to 0.30 mg/L by 1–2 mo. A gradual decline in median milk iron concentration continued through the following months, stabilizing at 0.20–0.21 mg/L by 6 mo (Figure 7A and B). The pooled median total iron intake from 1 to 6 mo was 0.20 mg/d.

### Comparison with published values

Although considering infant hepatic iron reserves at birth, the IOM has set the AI for iron from 0 to 6 mo at 0.27 mg/d based on a human milk iron concentration of 0.35 mg/L averaged from 9 small studies published between 1976 and 1993 [37]. A recent study of 32 healthy, exclusively breastfeeding Polish women whose milk was sampled at 4 to 6 wk postpartum found a median iron concentration of 0.33 mg/L (IQR: 0.26–0.46) [40]. This result is consistent with early lactation data from the MILQ study. Earlier data suggested a mean milk iron concentration of 0.30 mg/L during the first year of lactation, though the range of measured concentrations was wide (0.04–1.92 mg/L) [41]. Iron content was not measured in the MIREC study [9]. The longitudinal decline in human milk iron concentration is consistent with trends noted in the literature [39]. Aside from early lactation, milk iron concentrations in the MILQ study were lower than the IOM value used to set the AIs.

## Copper

### Background

Copper is involved in cellular respiration as a component of several metalloenzymes that act as oxidases to reduce molecular oxygen. Copper accumulates in the fetal liver during gestation and is mobilized during early infancy [42]. The copper concentration of human milk has been reported as highest in colostrum and transitional milk and decreasing throughout lactation [39]. Maternal copper intake and status do not affect milk copper concentrations [39].

### Results

**FIGURE 8:**
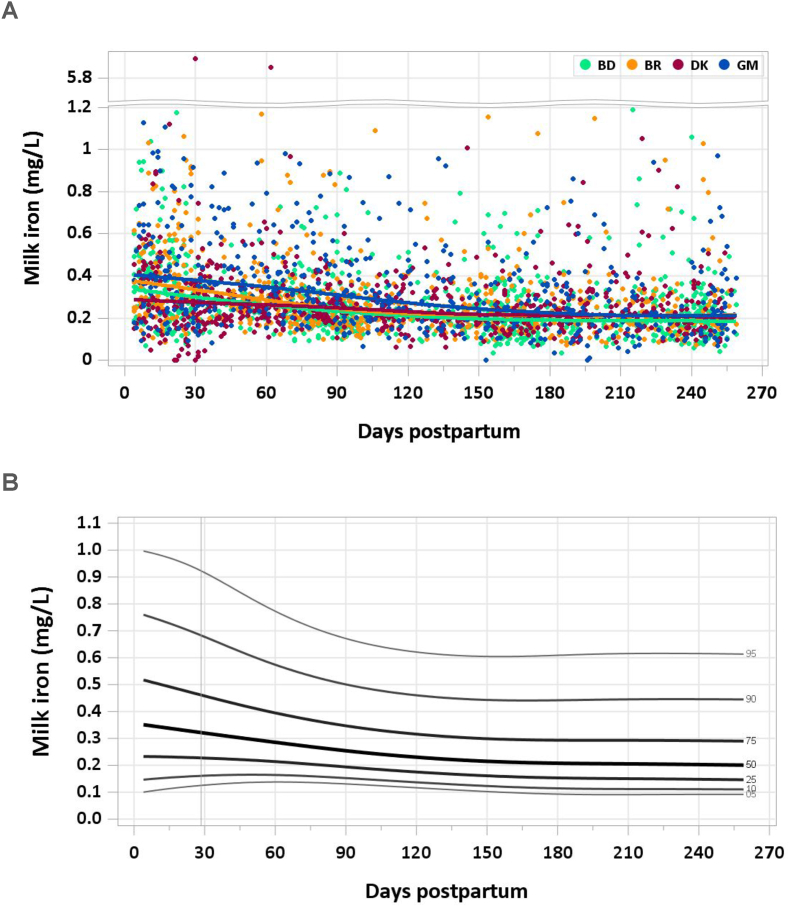
(A) Distribution of human milk copper concentrations. (B) Percentile curves for copper concentration in human milk.

In the MILQ study, 4 samples were excluded from data analysis because of implausible milk copper concentrations. No samples were excluded from E-MILQ. Median milk copper concentrations decreased from 0.52 mg/L at 4–17 d to 0.36 mg/L at 1–2 mo lactation. A gradual decline in milk copper concentration continued throughout the study period, with a median of 0.21 mg/L at 5–6 mo and 0.16 mg/L at >8 mo (Figure 8A and B). The pooled median total copper intake from 1 to 6 mo was 0.22 mg/d.

### Comparison with published values

Based on the results of 15 studies published between 1976 and 1998 in which the milk of 6–50 women was sampled, the IOM estimated the milk copper concentration to be 0.25 mg/L and set the AI for infants 0–6 mo at 0.20 mg/d [37]. The milk copper concentrations measured in the present study were on par with the IOM value. A recent multisite study of trace elements measured in human milk at an average of 7 mo postpartum found a mean copper concentration of 0.18 ± 0.07 mg/L, which agrees with MILQ copper concentrations at a similar point in lactation [43]. Likewise, the Canadian MIREC milk copper concentration from 3 to 10 wk [0.42 mg/L (0.43 μg/g)] [9] was consistent with MILQ values over a similar time period.

## Zinc

### Background

Zinc is a component of enzymes that maintain the structural integrity of proteins and regulate gene expression [37]. It is an essential nutrient for fetal and neonatal growth and brain development [44]. In addition to stunted growth, infant zinc deficiency is associated with compromised immune function and increased morbidity and mortality from diarrheal and respiratory disease [39]. Human milk zinc concentrations are refractory to maternal dietary intake, nutritional status, and supplementation [35,39].

### Results

**FIGURE 9:**
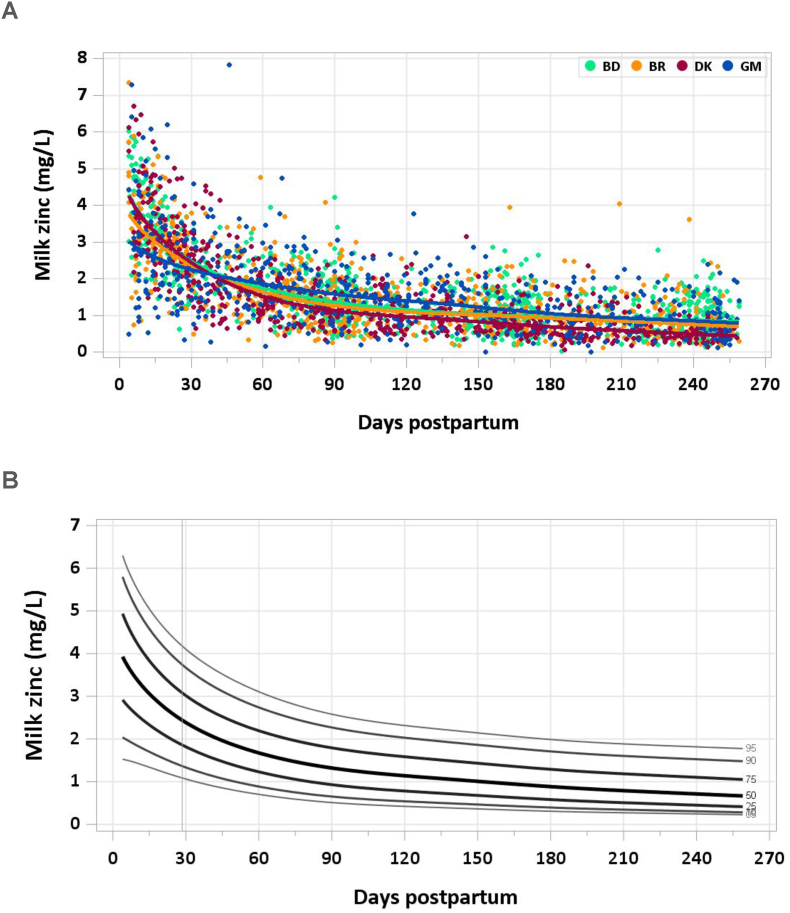
(A) Distribution of human milk zinc concentrations. (B) Percentile curves for zinc concentration in human milk.

No samples were excluded from the analysis for implausible milk zinc concentrations. Median zinc concentration declined from 3.33 mg/L at 4–17 d to 1.96 mg/L at 1–2 mo, 0.94 mg/L at 5–6 mo, and 0.68 mg/L by the end of the study period (Figure 9A and B). The pooled median total zinc intake from 1 to 6 mo was 1.1 mg/d.

### Comparison with published values

Although human milk zinc concentrations are recognized to decline from early lactation through 6 mo, the IOM has set the AI for zinc for 0–6 mo at 2.0 mg/d, calculated from an estimated milk zinc concentration of 2.5 mg/L and intake of 0.78 L/d [37]. According to the referenced study [45], this concentration is consistent with human milk zinc concentration at 1–2 mo postpartum and ensures adequacy, although it may exceed needs by 4–6 mo of age [37]. The downward trend in zinc concentration from early through mature milk observed in the MILQ study is consistent with the literature. Zinc concentrations measured in MILQ are aligned with the estimated mean zinc transfer to the infant of ∼1.75 mg/d at 1 mo and ∼0.7 mg/d at 6 mo reported in a detailed review of evidence on human milk zinc [46]. They are also consistent with data from the MIREC study, which found a median concentration of 1.78 mg/L (1.83 μg/g) at 3–10 wk [9]. The discrepancy between MILQ zinc results and the IOM concentration used to set the AIs can be explained by the decision of the IOM to set the value based on the early postpartum period when zinc concentrations are high.

## Iodine

### Background

Iodine is a component of thyroxine (T4) and triiodothyronine (T3), thyroid hormones regulating fundamental processes, including metabolism, fetal growth, and neurodevelopment [47]. Iodine deficiency is associated with impaired thyroid hormone synthesis [47,48]. Low thyroid hormone concentrations due to severe iodine deficiency in utero and during infancy affect physical, neurological, and intellectual development, but the effects of mild iodine deficiency are less clear [49]. Infants have high iodine requirements because of elevated thyroid hormone turnover [50]. Milk iodine concentration is strongly associated with maternal iodine intake [50], peaking within a few hours after consumption [51]. In many countries, diets lack sufficient iodine, and iodine deficiency is prevented by fortifying salt with iodine [52].

### Results

**FIGURE 10:**
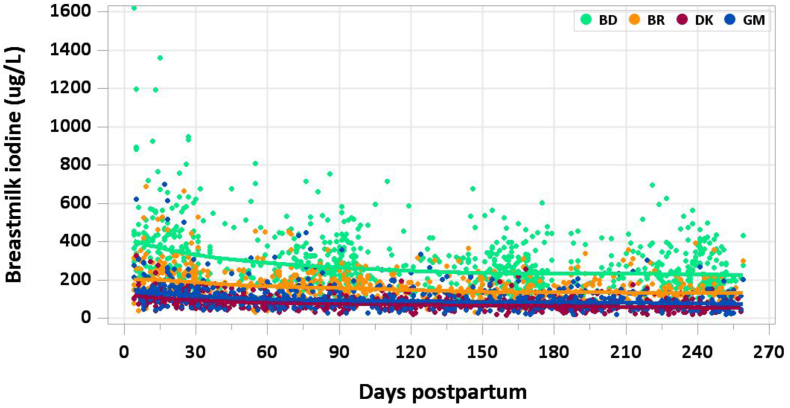
Distribution of human milk iodine concentrations.

A total of 3050 samples were analyzed (2477 MILQ, 573 E-MILQ), of which 3 were removed for implausible values (3 MILQ, 0 E-MILQ), and 322 for not meeting inclusion criteria (270 MILQ, 52 E-MILQ), for a total included sample size of 2725 (2204 MILQ, 521 E-MILQ). Milk iodine concentrations ranged from 113 μg/L in Denmark to 403 μg/L in Bangladesh at 4–17 d, decreased to 85 μg/L in Denmark and 270 μg/L in Bangladesh at 1–2 mo, and further modestly decreased throughout lactation within each site (Figure 10). Median milk iodine was consistently higher in Bangladesh than in other countries, likely related to the higher iodine intake of mothers. The decision was made not to generate RVs or percentile curves for iodine because of the wide variability caused by national differences in salt fortification policy.

### Comparison with published values

The AI for iodine in infants 0–6 mo of age is 110 μg/d, based on a milk iodine concentration of 146 μg/L obtained in a single study of 24 United States women [53] and a milk intake of 0.78 L/d [37]. The milk iodine concentration in Brazil was similar to the study referenced by IOM. Variations in milk iodine concentrations observed between study sites in the MILQ study reflect different iodine intake levels, likely partly attributed to differences in access to iodized salt.

A previous multicenter study of lactating women (*n* = 635) in 3 study sites with mandatory salt iodization reported a median milk iodine concentration of 171 μg/L (IQR: 123–235 μg/L) [54]. The MIREC study found a median concentration of 178 μg/L (183 μg/kg) in Canadian mothers at 3–10 wk [9]. Reviews of cross-sectional studies suggest that a median milk iodine concentration of 100–200 μg/L reflects adequate iodine status in lactating women, but data on the association of milk iodine concentration with maternal and infant thyroid function are limited [50]. We measured thyroid hormone parameters in mothers and infants (thyroglobulin, TSH, and total T4) and will report on the association with milk iodine concentration and iodine status in infants (urinary iodine concentration) in a separate paper.

## Selenium

### Background

Selenium is a component of selenoproteins necessary for thyroid hormone metabolism, which is critical for early development [55]. In human milk, selenium is a component of glutathione peroxidase and, to a lesser extent, selenocystamine, selenocystine, and selenomethionine [56]. Selenium levels are high in colostrum and fall with time during lactation. Given that maternal dietary intake reflects the selenium content of soils where foods are grown, geographical variations in maternal selenium intake and human milk concentrations are expected [39].

### Results

**FIGURE 11:**
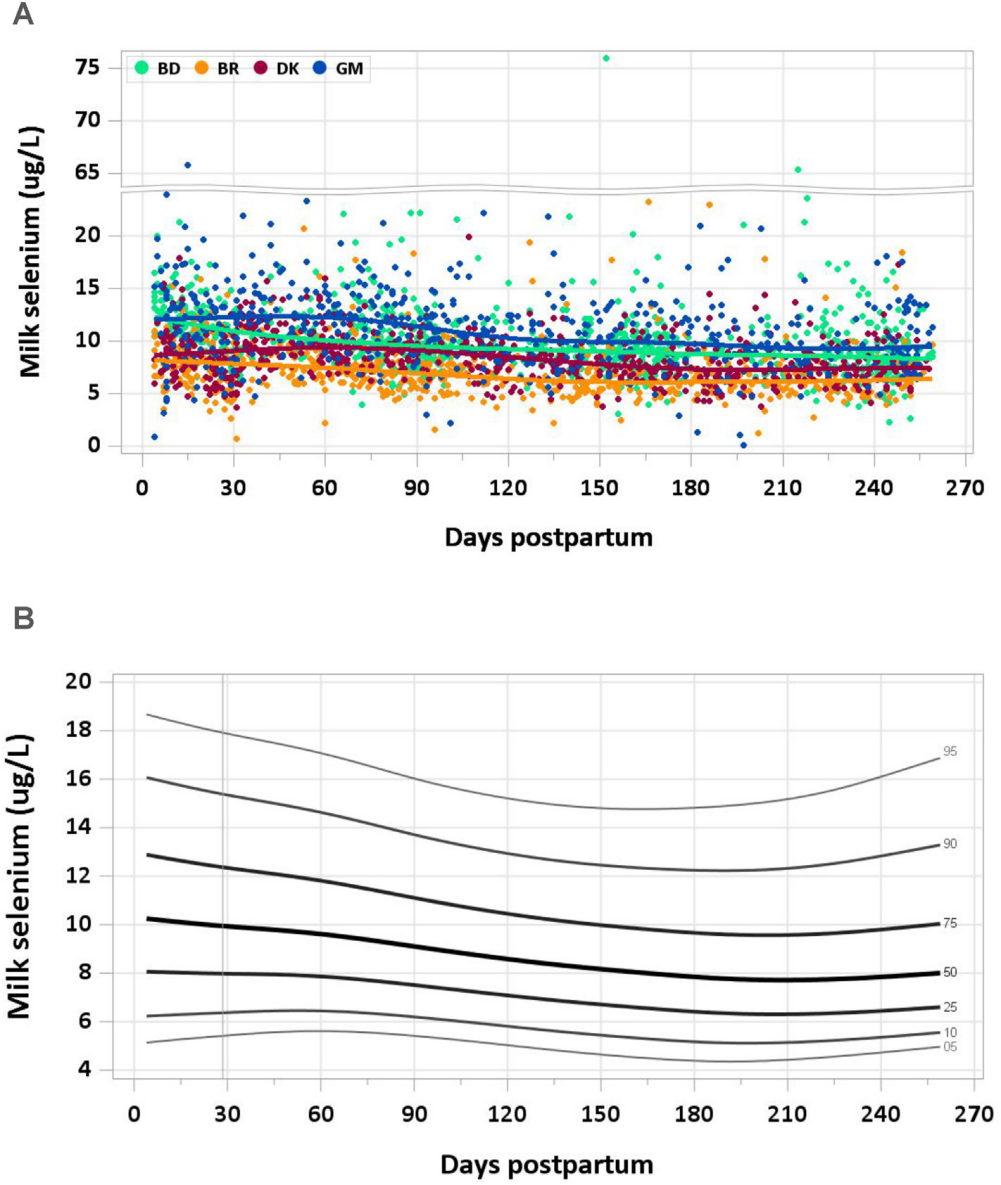
(A) Distribution of human milk selenium concentrations. (B) Percentile curves for selenium concentration in human milk.

No samples were removed from the analysis for implausible values. Median milk selenium concentration was 10.2 μg/L at 4–17 d, after which it decreased gradually to a plateau of 7.8–8.0 μg/L by 5–6 mo (Figure 11A and B). The pooled median total selenium intake from 1 to 6 mo was 7.3 μg/d.

### Comparison with published values

The IOM set the AI for selenium intake for infants 0–6 mo at 15 μg/d based on an average mature milk selenium concentration of 18 μg/L in well-nourished mothers and infant milk consumption of 0.78 L/d [37]. Studies referenced by the IOM were mainly carried out in the 1980s and found a selenium concentration of 33–80 μg/L in colostrum, followed by a substantial reduction to 18–29 μg/L by 1 wk postpartum. The average selenium concentration in mature milk has been reported as 10–23 μg/L. The IOM concentration of 18 μg/L was based on consideration of a subset of mature milk samples collected from United States and Canadian mothers, in which the range of milk selenium concentration was 15–20 μg/L [57].

The median milk selenium concentrations in the MILQ study are considerably lower than the IOM RVs from colostrum through mature milk. The selenium concentration in MIREC was 19.4 μg/L (20 ng/g) [9], which is more similar to the IOM value and substantially higher than in the MILQ study (8 μg/L). Given the strict validation of our method to ensure accurate results [6], these differences may be due to maternal dietary intake, which depends on regional soil selenium content [58]. Dietary intake of organic selenium has been considered a determinant of human milk selenium concentration in most but not all studies [[59], [60], [61]].

## Discussion

The primary objective of the MILQ study was to develop RVs for nutrient concentrations in the milk of healthy mothers during the first 8.5 mo of lactation. The mineral concentrations in human milk are similar among the 4 study populations except for calcium and iodine. Previous literature suggests that minerals in milk are relatively independent of maternal intake, with the exception of calcium, selenium, and iodine.

Despite limitations in previously available data, results from the MILQ study corroborated IOM milk nutrient concentrations for magnesium, calcium, and copper; for sodium, zinc, and iron concentrations early in lactation; and for potassium concentrations later in lactation. MILQ data suggest that IOM values may underestimate milk mineral concentrations of phosphorus. Selenium concentrations in the MILQ study are substantially lower than IOM estimates. Explanations for this discrepancy may include differences in maternal selenium intake. Milk iodine concentrations were measured in the MILQ study but varied between countries due to national differences in salt fortification policy, so comparison with the IOM value was not undertaken.

In the MILQ study, milk volume quantified by deuterium oxide dose-to-mother (or test weighing in Denmark) and milk nutrient concentration enabled the calculation of total nutrient intake by the infant at multiple time points over the first 8.5 mo of lactation. The pooled median infant intake of most minerals from 1 to 6 mo was within 20% of the AI established by the IOM for this time period. Exceptions were phosphorus (125% of the AI), zinc (55% of the AI, which can be explained by the IOM’s selection of 1–2 mo postpartum as the reference period), and selenium (49% of the AI).

In conclusion, the use of a dual-detector equipped ICP-MS enabled simultaneous quantification of multiple minerals in a small volume of human milk to produce estimated percentiles for the concentrations of minerals. MILQ data suggest that IOM RVs may underestimate milk concentrations of potassium in early lactation and phosphorus through 8.5-mo lactation while overestimating concentrations of zinc, iron, and sodium in later lactation and selenium through 8.5-mo lactation. The data presented here can be used to evaluate the adequacy of milk mineral concentrations and the impact of maternal supplementation. It is hoped that where concentrations differ substantially from values used to set current AIs for infants, those will be improved and used to set Estimated Average Requirements (EARs).

## Author contributions

The authors’ responsibilities were as follows – LHA, MMI, GK, KFM, SEM, SS-F, DH: designed research; AMD, SS-F, DdBM, GTS, DH: conducted research; DH, MA, JMP: analyzed data; DKD, LHA, MA: wrote the paper; LHA: primary responsibility for the final content; and all authors: read and approved the final manuscript.

## Data availability

Data described in the manuscript, code book, and analytic code will be available upon request, pending application and approval.

## The MILQ Study Consortium

Lindsay H. Allen, Sophie E. Moore, Gilberto Kac, Kim F. Michaelsen, Christian Mølgaard, M. Munirul Islam, Maria Andersson, Setareh Shahab-Ferdows, Sophie H. Christensen, Jack I. Lewis, Janet M. Peerson, Xiuping Tan, Daphna K. Dror, Andrew M. Doel, Daniela de Barros Mucci, Bruna C. Schneider, Farhana Khanam, Adriana Divina de Souza Campos, Gabriela Torres Silva, Fanta Nije, Mehedi Hassan, Amanda C. Figueiredo, Daniela Hampel.

## Funding

This study was supported by the Gates Foundation (OPP1148405/INV-002300, OPP1061055) and USDA intramural funds (2023-51530-025-00D).

## Conflict of interest

The authors report no conflicts of interest.
