# Supplementary material for: Reference Values for Minerals in Human Milk: the Mothers, Infants and Lactation Quality (MILQ) Study
Source: Adv Nutr. 2025 Aug 26;16(Suppl 1):100431. doi: 10.1016/j.advnut.2025.100431 (PMC12592237; doi:10.1016/j.advnut.2025.100431)
Supplement: multimedia component 1 [file mmc1.docx]

**Supplementary Table 1.** Monthly percentile summaries for mineral concentration in human milk

| Sodium (mg/L) | **Age** | **P05** | **P10** | **P25** | **P50** | **P75** | **P90** | **P95** |
| --- | --- | --- | --- | --- | --- | --- | --- | --- |
|  |  |  |  |  |  |  |  |  |
|  | 4-17 d | 117.8 | 136.6 | 170.8 | 219.9 | 303.7 | 473.6 | 696.5 |
|  | 18-31 d | 95.4 | 109.2 | 134.8 | 172.3 | 236.9 | 365.3 | 528.1 |
|  | 1-2 m | 78.4 | 89.4 | 109.8 | 139.8 | 191.2 | 292.2 | 419.0 |
|  | 2-3 m | 67.5 | 77.3 | 95.2 | 121.0 | 164.4 | 249.6 | 357.5 |
|  | 3-4 m | 60.0 | 69.7 | 86.9 | 110.7 | 150.2 | 228.1 | 329.2 |
|  | 4-5 m | 53.2 | 62.9 | 79.7 | 102.3 | 139.2 | 212.9 | 311.0 |
|  | 5-6 m | 48.3 | 58.0 | 74.5 | 96.5 | 132.2 | 204.2 | 302.3 |
|  | 6-7 m | 46.4 | 55.9 | 72.2 | 94.1 | 129.5 | 201.7 | 301.2 |
|  | 7-8 m | 46.3 | 55.5 | 71.6 | 93.4 | 129.2 | 201.8 | 302.3 |
|  | 8-8.5 m | 46.7 | 55.6 | 71.5 | 93.4 | 129.4 | 202.5 | 303.5 |
|  |  |  |  |  |  |  |  |  |
|  |  |  |  |  |  |  |  |  |
| Potassium (mg/L) | **Age** | **P05** | **P10** | **P25** | **P50** | **P75** | **P90** | **P95** |
|  |  |  |  |  |  |  |  |  |
|  | 4-17 d | 430 | 501 | 590 | 666 | 739 | 813 | 868 |
|  | 18-31 d | 412 | 473 | 551 | 621 | 688 | 758 | 812 |
|  | 1-2 m | 399 | 452 | 521 | 584 | 647 | 716 | 768 |
|  | 2-3 m | 388 | 434 | 496 | 555 | 616 | 682 | 735 |
|  | 3-4 m | 379 | 422 | 481 | 538 | 597 | 663 | 715 |
|  | 4-5 m | 371 | 413 | 471 | 527 | 586 | 652 | 703 |
|  | 5-6 m | 363 | 405 | 463 | 520 | 579 | 644 | 696 |
|  | 6-7 m | 355 | 398 | 457 | 514 | 573 | 639 | 689 |
|  | 7-8 m | 349 | 392 | 451 | 509 | 568 | 633 | 683 |
|  | 8-9 m | 344 | 387 | 447 | 505 | 564 | 629 | 679 |
|  |  |  |  |  |  |  |  |  |
|  |  |  |  |  |  |  |  |  |
| Magnesium (mg/L) | **Age** | **P05** | **P10** | **P25** | **P50** | **P75** | **P90** | **P95** |
|  |  |  |  |  |  |  |  |  |
|  | 4-17 d | 16.4 | 19.8 | 24.6 | 29.4 | 34.2 | 39.3 | 43.1 |
|  | 18-31 d | 17.5 | 20.5 | 24.9 | 29.2 | 33.7 | 38.4 | 41.9 |
|  | 1-2 m | 19.1 | 21.8 | 25.9 | 30.1 | 34.4 | 38.9 | 42.1 |
|  | 2-3 m | 20.8 | 23.6 | 27.6 | 31.8 | 36.0 | 40.5 | 43.6 |
|  | 3-4 m | 21.7 | 24.5 | 28.7 | 32.9 | 37.3 | 41.7 | 44.8 |
|  | 4-5 m | 21.8 | 24.7 | 28.9 | 33.3 | 37.7 | 42.1 | 45.1 |
|  | 5-6 m | 21.5 | 24.4 | 28.7 | 33.1 | 37.5 | 41.9 | 45.0 |
|  | 6-7 m | 20.9 | 23.8 | 28.1 | 32.6 | 37.1 | 41.5 | 44.5 |
|  | 7-8 m | 20.3 | 23.2 | 27.5 | 32.0 | 36.5 | 41.0 | 43.9 |
|  | 8-8.5 m | 19.8 | 22.7 | 27.1 | 31.6 | 36.1 | 40.5 | 43.5 |
|  |  |  |  |  |  |  |  |  |
|  |  |  |  |  |  |  |  |  |
| Phosphorus (mg/L) | **Age** | **P05** | **P10** | **P25** | **P50** | **P75** | **P90** | **P95** |
|  |  |  |  |  |  |  |  |  |
|  | 4-17 d | 94 | 115 | 142 | 167 | 190 | 214 | 231 |
|  | 18-31 d | 95 | 115 | 140 | 163 | 185 | 208 | 224 |
|  | 1-2 m | 96 | 114 | 138 | 159 | 179 | 199 | 214 |
|  | 2-3 m | 97 | 113 | 134 | 154 | 172 | 190 | 204 |
|  | 3-4 m | 97 | 112 | 132 | 150 | 167 | 185 | 197 |
|  | 4-5 m | 95 | 110 | 129 | 147 | 164 | 182 | 194 |
|  | 5-6 m | 93 | 107 | 127 | 144 | 162 | 179 | 192 |
|  | 6-7 m | 90 | 104 | 123 | 141 | 158 | 177 | 190 |
|  | 7-8 m | 88 | 101 | 119 | 137 | 155 | 174 | 188 |
|  | 8-8.5 m | 86 | 98 | 117 | 135 | 153 | 172 | 186 |
|  |  |  |  |  |  |  |  |  |
|  |  |  |  |  |  |  |  |  |
| Calcium (mg/L) | **Age** | **P05** | **P10** | **P25** | **P50** | **P75** | **P90** | **P95** |
|  |  |  |  |  |  |  |  |  |
|  | 4-17 d | 171 | 202 | 247 | 290 | 332 | 375 | 406 |
|  | 18-31 d | 175 | 206 | 249 | 292 | 334 | 376 | 406 |
|  | 1-2 m | 179 | 210 | 253 | 294 | 336 | 378 | 407 |
|  | 2-3 m | 182 | 211 | 253 | 294 | 334 | 375 | 404 |
|  | 3-4 m | 180 | 209 | 249 | 288 | 327 | 366 | 394 |
|  | 4-5 m | 176 | 204 | 242 | 279 | 316 | 353 | 379 |
|  | 5-6 m | 171 | 197 | 233 | 268 | 303 | 338 | 363 |
|  | 6-7 m | 165 | 189 | 223 | 256 | 289 | 322 | 346 |
|  | 7-8 m | 159 | 182 | 214 | 245 | 276 | 307 | 329 |
|  | 8-8.5 m | 154 | 176 | 207 | 237 | 266 | 296 | 317 |
|  |  |  |  |  |  |  |  |  |
|  |  |  |  |  |  |  |  |  |
| Iron (mg/L) | **Age** | **P05** | **P10** | **P25** | **P50** | **P75** | **P90** | **P95** |
|  |  |  |  |  |  |  |  |  |
|  | 4-17 d | 0.108 | 0.151 | 0.232 | 0.343 | 0.502 | 0.741 | 0.982 |
|  | 18-31 d | 0.122 | 0.159 | 0.229 | 0.326 | 0.470 | 0.697 | 0.938 |
|  | 1-2 m | 0.134 | 0.164 | 0.221 | 0.301 | 0.423 | 0.621 | 0.839 |
|  | 2-3 m | 0.135 | 0.159 | 0.203 | 0.268 | 0.368 | 0.532 | 0.714 |
|  | 3-4 m | 0.123 | 0.144 | 0.183 | 0.240 | 0.328 | 0.476 | 0.640 |
|  | 4-5 m | 0.108 | 0.129 | 0.167 | 0.221 | 0.305 | 0.449 | 0.610 |
|  | 5-6 m | 0.096 | 0.117 | 0.156 | 0.210 | 0.295 | 0.441 | 0.606 |
|  | 6-7 m | 0.091 | 0.112 | 0.151 | 0.206 | 0.293 | 0.444 | 0.613 |
|  | 7-8 m | 0.092 | 0.111 | 0.149 | 0.204 | 0.292 | 0.446 | 0.616 |
|  | 8-8.5 m | 0.092 | 0.111 | 0.147 | 0.201 | 0.291 | 0.445 | 0.614 |
|  |  |  |  |  |  |  |  |  |
|  |  |  |  |  |  |  |  |  |
| Copper (mg/L) | **Age** | **P05** | **P10** | **P25** | **P50** | **P75** | **P90** | **P95** |
|  |  |  |  |  |  |  |  |  |
|  | 4-17 d | 0.335 | 0.386 | 0.460 | 0.537 | 0.620 | 0.714 | 0.788 |
|  | 18-31 d | 0.260 | 0.301 | 0.363 | 0.429 | 0.502 | 0.586 | 0.654 |
|  | 1-2 m | 0.206 | 0.241 | 0.296 | 0.355 | 0.421 | 0.500 | 0.564 |
|  | 2-3 m | 0.161 | 0.192 | 0.241 | 0.295 | 0.357 | 0.432 | 0.495 |
|  | 3-4 m | 0.132 | 0.160 | 0.205 | 0.256 | 0.316 | 0.389 | 0.452 |
|  | 4-5 m | 0.112 | 0.138 | 0.179 | 0.228 | 0.285 | 0.358 | 0.420 |
|  | 5-6 m | 0.096 | 0.120 | 0.159 | 0.205 | 0.261 | 0.333 | 0.395 |
|  | 6-7 m | 0.084 | 0.106 | 0.143 | 0.187 | 0.241 | 0.312 | 0.374 |
|  | 7-8 m | 0.074 | 0.095 | 0.129 | 0.172 | 0.224 | 0.293 | 0.355 |
|  | 8-8.5 m | 0.067 | 0.087 | 0.120 | 0.161 | 0.212 | 0.280 | 0.341 |
|  |  |  |  |  |  |  |  |  |
|  |  |  |  |  |  |  |  |  |
| Zinc (mg/L) | **Age** | **P05** | **P10** | **P25** | **P50** | **P75** | **P90** | **P95** |
|  |  |  |  |  |  |  |  |  |
|  | 4-17 d | 1.421 | 1.835 | 2.552 | 3.382 | 4.239 | 5.029 | 5.510 |
|  | 18-31 d | 1.160 | 1.461 | 1.989 | 2.607 | 3.281 | 3.964 | 4.409 |
|  | 1-2 m | 0.851 | 1.070 | 1.470 | 1.960 | 2.522 | 3.114 | 3.513 |
|  | 2-3 m | 0.591 | 0.749 | 1.055 | 1.467 | 1.959 | 2.465 | 2.801 |
|  | 3-4 m | 0.458 | 0.585 | 0.841 | 1.213 | 1.669 | 2.126 | 2.422 |
|  | 4-5 m | 0.389 | 0.498 | 0.723 | 1.065 | 1.498 | 1.934 | 2.217 |
|  | 5-6 m | 0.330 | 0.423 | 0.621 | 0.935 | 1.348 | 1.772 | 2.051 |
|  | 6-7 m | 0.285 | 0.363 | 0.536 | 0.826 | 1.224 | 1.643 | 1.922 |
|  | 7-8 m | 0.256 | 0.322 | 0.472 | 0.743 | 1.134 | 1.555 | 1.841 |
|  | 8-8.5 m | 0.234 | 0.292 | 0.428 | 0.685 | 1.072 | 1.498 | 1.791 |
|  |  |  |  |  |  |  |  |  |
|  |  |  |  |  |  |  |  |  |
| Selenium (measured as oxide) (μg/L) | **Age** | **P05** | **P10** | **P25** | **P50** | **P75** | **P90** | **P95** |
|  |  |  |  |  |  |  |  |  |
|  | 4-17 d | 5.22 | 6.27 | 8.04 | 10.16 | 12.73 | 15.87 | 18.46 |
|  | 18-31 d | 5.37 | 6.35 | 7.99 | 9.99 | 12.44 | 15.48 | 18.03 |
|  | 1-2 m | 5.56 | 6.43 | 7.93 | 9.77 | 12.06 | 14.97 | 17.46 |
|  | 2-3 m | 5.53 | 6.33 | 7.69 | 9.35 | 11.44 | 14.15 | 16.52 |
|  | 3-4 m | 5.21 | 5.99 | 7.27 | 8.81 | 10.73 | 13.26 | 15.54 |
|  | 4-5 m | 4.80 | 5.60 | 6.86 | 8.34 | 10.17 | 12.63 | 14.94 |
|  | 5-6 m | 4.48 | 5.27 | 6.53 | 7.98 | 9.79 | 12.31 | 14.78 |
|  | 6-7 m | 4.38 | 5.13 | 6.33 | 7.75 | 9.59 | 12.26 | 15.00 |
|  | 7-8 m | 4.60 | 5.26 | 6.38 | 7.78 | 9.69 | 12.60 | 15.70 |
|  | 8-8.5 m | 4.87 | 5.47 | 6.54 | 7.94 | 9.94 | 13.10 | 16.56 |

**Supplementary Table 2**. Median infant mineral intake by study visit

|  | **1-3.49 mo** | **3.5-5.99 mo** | **6-8.5 mo** |
| --- | --- | --- | --- |
| **Sodium (mg/d)** | 102.70 | 83.21 | 65.01 |
| **Potassium (mg/d)** | 438.80 | 445.10 | 351.10 |
| **Magnesium (mg/d)** | 24.74 | 27.96 | 21.61 |
| **Phosphorus (mg/d)** | 122.30 | 123.00 | 96.78 |
| **Calcium (mg/d)** | 235.00 | 231.10 | 165.70 |
| **Iron (mg/d)** | 0.22 | 0.187 | 0.138 |
| **Copper (mg/d)** | 0.244 | 0.183 | 0.114 |
| **Zinc (mg/d)** | 1.21 | 0.871 | 0.552 |
| **Selenium (μg/d)** | 7.746 | 6.89 | 5.17 |
